# Receptor-Based Virtual Screening of EGFR Kinase Inhibitors from the NCI Diversity Database

**DOI:** 10.3390/molecules15064041

**Published:** 2010-06-04

**Authors:** Kiattawee Choowongkomon, Orathai Sawatdichaikul, Napat Songtawee, Jumras Limtrakul

**Affiliations:** 1 Department of Biochemistry, Kasetsart University, Bangkok, 10900, Thailand; E-Mails: lukmoo86@hotmail.com (O.S.); napat_s@hotmail.com (N.S.); 2 Department of Chemistry, Kasetsart University, Bangkok 10900, Thailand; E-Mail: fscijrl@ku.ac.th (J.L.)

**Keywords:** EGFR-TK, anti-cancer drugs, NCI diversity set, virtual screening, docking

## Abstract

Epidermal growth factor receptor (EGFR) abnormalities have been associated with several types of human cancer. The crystal structures of its tyrosine kinase domain (EGFR-TK) complexed with small molecule inhibitors revealed the kinase inhibition modes, prompting us to search for novel anti-cancer drugs. A total of 1,990 compounds from the National Cancer Institute (NCI) diversity set with nonredundant structures have been tested to inhibit cancer cell lines with unknown mechanism. Cancer inhibition through EGFR-TK is one of the mechanisms of these compounds. In this work, we performed receptor-based virtual screening against the NCI diversity database. Using two different docking algorithms, AutoDock and Gold, combined with subsequent post-docking analyses, we found eight candidate compounds with high scoring functions that all bind to the ATP-competitive site of the kinase. None of these compounds belongs to the main group of the currently known EGFR-TK inhibitors. Binding mode analyses revealed that the way these compounds complexed with EGFR-TK differs from quinazoline inhibitor binding and the interaction mainly involves hydrophobic interactions. Also, the common kinase-inhibitor (NH---N and CO---HC) hydrogen bonds between the hinge region and the hit compounds are rarely observed. Our results suggest that these molecules could be developed as novel lead compounds in anti-cancer drug design.

## 1. Introduction

The receptor tyrosine kinase (RTK) of the epidermal growth factor receptor (EGFR, ErbB) family plays a crucial role in cellular signaling pathways that regulate key functions such as differentiation, proliferation, survival and apoptosis [1]. This family consists of four conserved structural members: epidermal growth factor receptor (EGFR)/HER1/ErbB1, ErbB2/HER2/c-neu, ErbB3/HER3, and ErbB4/HER4). They all share a common domain organization which is composed of a ligand-binding extracellular domain, a hydrophobic transmembrane segment, and an intracellular part which includes a juxtamembrane domain (53 aa) [2], a tyrosine kinase (TK) domain (~260 aa), and a C-terminal tyrosine-rich region (~232 aa) [3]. Upon binding of the growth factors, including epidermal growth factor (EGF) and transforming growth factor-α (TGF-α), to the extracellular part of the receptor, the growth factors induce the homo- and/or heterodimerization of the receptor, activate the TK domain to phosphorylate at its C-terminal tail, and eventually, initiate downstream signaling pathways [4].

**Figure 1:**
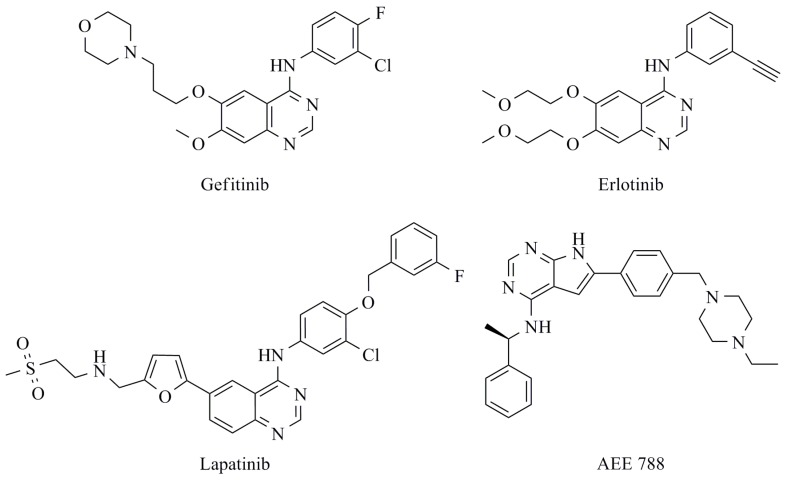
EGFR-TK-selective inhibitors; gefitinib, erlotinib and AEE788 and EGFR/HER2-TK-dual inhibitor; lapatinib.

Overexpression of EGFR is observed in approximately 60% of patients with non-small cell lung cancer (NSCLC), which is the largest subset of lung cancer and the major cause of cancer death around the world [5,6,7]. Therefore, the deregulation of EGFR has been clinically implicated as a target for NSCLC therapy [8]. At present, there are four classes of anti-EGFR agents used for cancer therapy: (i) monoclonal antibodies (MAbs) that is directed against the extracellular domain of EGFR [9,10], (ii) antisense oligonucleotides that inhibit EGFR synthesis [11], (iii) antibody-based immunoconjugates [12,13] and (iv) small molecules that block the kinase activity [also known as tyrosine kinase inhibitors (TKIs)]. All of them have been applied clinically to cancer therapy involving EGFR. These small molecules, compete to ATP at the TK domain, include the first clinically used gefitinib (Iressa™, AstraZeneca), erlotinib (Tarceva™, OSI-Pharma/Genentech/Roche), and lapatinib (Tykerb™, GlaxoSmithKline) [14] (Figure 1). Although gefitinib-susceptible cases are observed in a small number of NSCLC patients, sensitive response to treatment with gefitinib could be associated with several mutations in the TK domain of EGFR, such as the L834R mutation which is the hot spot mutation in NSCLC patients that enhances kinase activity. However, there is evidence that the acquired clinical drug resistance to gefitinib and erlotinib resulting in the T766M mutation can be observed in NSCLC patients who carry the primary cause L834R mutation [15,16].

**Figure 2:**
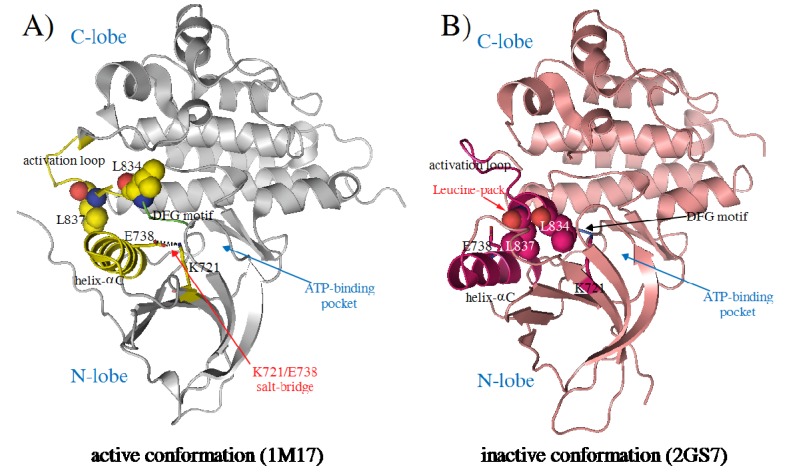
The ribbon presentation of EGFR-TK structures in the active conformation (A) and the inactive conformation (B). Both structures are different in the arrangement of the activation loop (A-loop) and the orientation of the helix-αC.

So far, twenty-six crystal structures of both wild-type and mutant EGFR-TK have been reported [17,18,19,20,21,22]. These structures revealed active and inactive conformations of EGFR-TK, which differ in α helix-C orientation activation loop (A-loop) arrangement DGF motif, and L834 and L837 (Figure 2). The crystal structures of EGFR-TK complexed with the drugs erlotinib and gefitinib have been used to explore key binding modes including the hydrogen bonds between the kinase hinge region and the quinazoline moiety of the drugs [17,20]. These studies help us understand the interaction between EGFR-TK and inhibitors, useful for the structural-based design of novel kinase inhibitors.

Receptor-based virtual screening (VS), a high throughput computational drug discovery approach, has become a potentially powerful and inexpensive method for searching novel lead compounds in drug development [23]. Receptor-based VS is based on a molecular docking technique requiring knowledge of the three-dimensional structure of the target protein binding site and their likelihood to bind to proteins [24]. Molecular docking-based VS techniques have been successful in searching for novel inhibitors in several cases, such as BCR-ABL tyrosine kinase [25], Chk1 [26], FKBP [27], as well as protein tyrosine phosphatases (PTP) [28,29]. However, a few receptor-based virtual screenings of EGFR-TK and/or HER2-TK against commercial and in-house chemical compounds in the series of anilinoquinazolines, pyridopyrimidines and pyrrolopyrimidines have been reported [30,31,32,33,34,35].

In this paper, we report the molecular docking-based virtual screening of EGFR-TK against the compound diversity set of the National Cancer Institute (NCI) Database. The NCI diversity set contains 1,990 compounds with nonredundant structures, all of which have been derived from plant sources since 1960 [36]. This diversity set was assessed for anti-cancer activity in several solid tumor lines such as leukemia, NSCLC, colon, melanoma and ovarian cell lines. Using different molecular docking techniques combined with post-docking analyses, we found eight compounds with high rank scoring functions and their binding modes to EGFR-TK were predicted.

## 2. Results and Discussion

**Figure 3:**
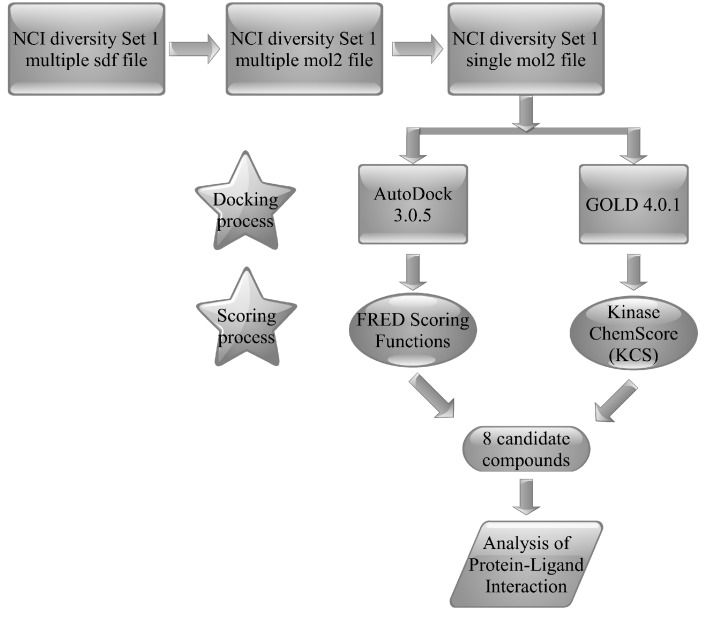
Schematic diagram of the molecular docking-based virtual screening workflow.

EGFR-TK is an attractive target for cancer therapy. Although several inhibitors of EGFR-TK have been clinically validated for the treatment of patients with NSCLC and breast cancer during the past several years [37], the search for new active compounds against EGFR-TK is still considerably challenging. Our work aimed at using high-throughput molecular docking to filter 1,990 NCI diversity compounds to identify new anti-EGFR inhibitors. A schematic representation of our workflow is shown in Figure 3. 

Recently, receptor-based virtual screening on EGFR-TK against 128 anilinoquinazoline analogues has been carried out using the crystal structures of erlotinib − EGFR-TK (PDB: 1M17) as a search model [32]. The 1M17 structure indicates a water-mediated hydrogen bond for Thr766 – OH ∙∙∙ N3 of quinazoline moiety and this VS study demonstrated that the lack of water molecules in the binding pocket of EGFR-TK leads to incorrect results [32], consistent with the other VS report [30]. Nevertheless, we examined all available crystal structures of EGFR-TK and found that either not all structures contain the water molecules in the binding pocket or they are located in different positions out of the binding pocket. Whether the water molecules exist in the EGFR-TK binding site depends on the conformation of the bound ligand. Recent molecular dynamics studies have demonstrated that several water molecules are occupying that site, but not in the same position, indicating that they are not involved in primary ligand binding [38]. Moreover, our preliminary study of erlotinib self-docking to EGFR-TK without any water molecule showed the pose orientation of erlotinib is similar to that observed in the crystal structure (PDB: 1M17) with RMSD of 1.74 Å (Table S1). Therefore, we decided not to include water molecules as a part of the protein model in our docking procedures.

We have performed the high-throughput molecular docking by using AutoDock (combined with FRED calculation) and GOLD programs for EGFR-TK against the 1,990 NCI diversity compounds. The two programs use different algorithms for conformational searches: force field genetic algorithm for AutoDock and Empirical score genetic algorithm for GOLD. The recent docking study has reported the reliability of the kinase scoring function (KCS) of the GOLD docking for virtual screening of EGFR-TK to identify the new EGFR inhibitors [34]. Here, our dockings revealed eight compounds with high scoring are consensus between these two programs (Table1). The compound H, I, J, K and M were shown to be sensitive to the most cancer cell lines (supplementary data S1).

**Table 1:** The KSC score, differential GOLD scores and the estimated binding free energy of the eight lead compounds from GOLD docking and their average GI_50_.

Compounds	NSC no.	KCS score fitness value	GOLD Chemscore			ΔG(kJ/mol)	Average GI_50_^† ^(μg/mL)
			Hbond^§^	Lipo^‡^	DE Clash^٭^		
M	402959	41.20	-	334.07	2.18	-44.57	5.50 × 10^-5^
L	351123	35.05	-	308.58	0.72	-36.55	9.02 × 10^-5^
H	130813	34.04	0.97	257.89	0.03	-34.46	1.94 × 10^-6^
J	299137	32.39	0.97	212.94	0.82	-34.89	7.03 × 10^-5^
I	135371	32.28	-	357.52	11.93	-44.49	3.95 × 10^-5^
B	48283	32.08	-	264.37	0.30	-33.85	9.76 × 10^-5^
K	306698	30.24	0.78	236.62	4.21	-35.78	3.25 × 10^-6^
G	125910	29.20	2.86	188.34	2.34	-32.77	9.86 × 10^-5^

^†^The average GI_50_ overall cell line from NCI Cancer Screen Current Data, May 2009. The GI_50_ means drug concentration causing 50% cell-growth inhibition; ^§^ and ^‡^ refer to protein-ligand hydrogen-bond and lipophilic contribution to the chemscore values, respectively, and ^٭^ refer to protein-ligand clash penalty to the chemscore value.

**Figure 4:**
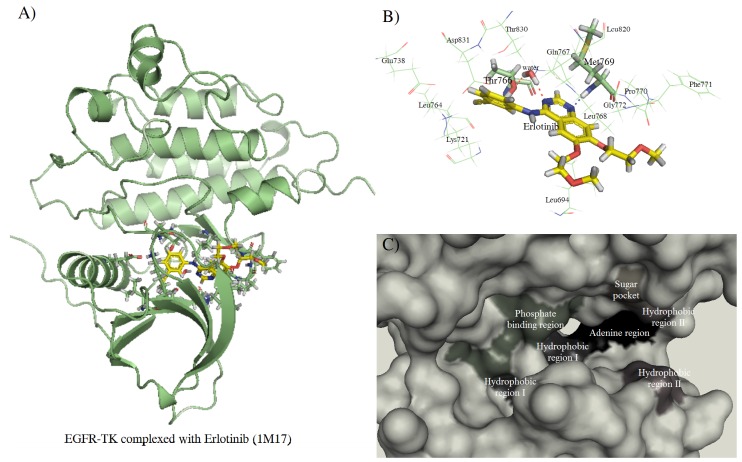
The complexes of EGFR-TK and erlotinib. (A) Overall structure of TK complexed with erlotinib. (B) The erlotinib and binding residues of kinase domain. (C) The molecular surface representation of the ATP-binding region which consists of adenine region, hydrophobic region I and II, sugar pocket and phosphate binding region.

The structure of the EGFR-TK complexed with erlotinib (Figure 4A) revealed that hydrophobic interaction is the main force whereas electrostatic interaction contributes to only to some extent. Besides the water-mediated hydrogen bond as discussed above, two significant hydrogen bonds include *(i)* Met769 – NH ∙∙∙ N1 quinazoline [17] and *(ii)* Gln767 - CO ∙∙∙ HC2 quinazoline [32] as shown in Figure 4B. Interaction between the hinge region (Thr766-Met769) and the bound ligands are highly conserved among protein kinases [39]. According to the pharmacophore model of the ATP-binding pocket of EGFR, five regions conserved throughout the protein kinases are classifiable. These include adenine region, hydrophobic region I and II, phosphate binding region, and sugar pocket as shown in Figure 4C [40]. The aniline moiety of erlotinib is inserted into the hydrophobic pocket of Val702, Met742, and Leu764, denoted as hydrophobic region I. Other hydrophobic residues such as Leu694, Leu768, Pro770, Phe771, and Leu820 in hydrophobic region II also contribute in the protein-inhibitor interaction. Moreover, there are two acidic residues (Glu738 and Asp831) located on the helix-αC and a phosphate binding region along the sugar pocket whereas the basic residue Lys721 located near the phosphate binding region, formed the salt-bridge to Glu738 in the helix-αC.

**Figure 5:**
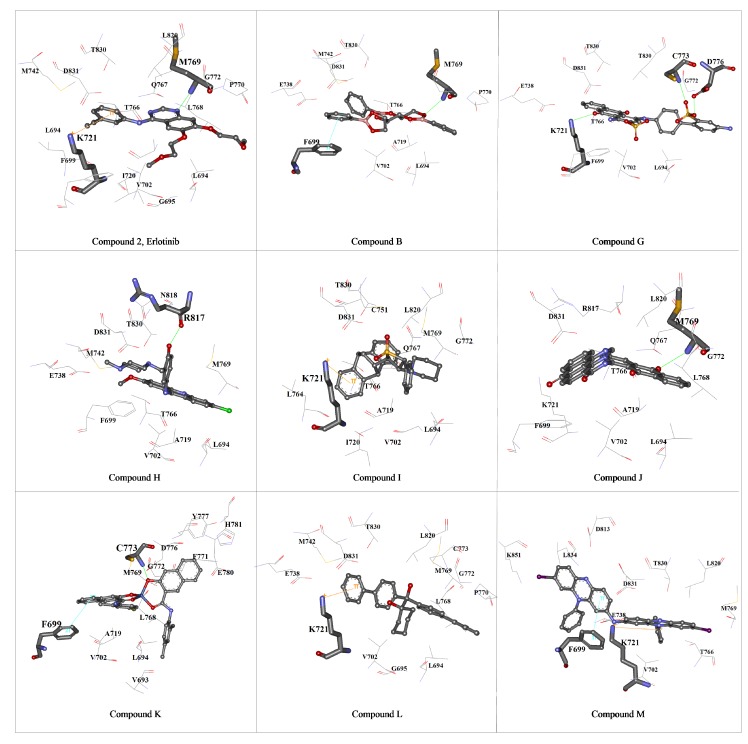
The 3D diagrams showing the interaction between the EGFR-TK and the eight hit compounds using Discovery Studio 2.5 (Accelrys Inc., CA, USA). The hit compounds, the amino acid residue interacting with the compounds and the other residues around the binding pocket are presented in *ball and stick*, *stick* and *line*, respectively. Hydrogen bond, π−π, and cation-π interactions between the compounds and the binding residues are shown as green solid, light blue and orange lines, respectively.

The interaction mode of EGFR-TK with these eight high rank molecules was analyzed by Ligand Interaction module in Discovery Studio 2.5 (Accelrys Inc., San Diego, CA, USA) as shown in Figure 5. The docking results revealed that the main interaction force of the candidate compounds with the EGFR-TK active site is hydrophobic (see below). All of the eight compounds contains aromatic ring and none of them was classified in the main three groups (anilinoquinazolines, pyrido-pyrimidines and pyrrolo-pyrimidines) of the known TK inhibitors. The important residues in the hydrophobic regions that interact with the hit compounds are Phe699, Leu764, Ile765, Val702, Leu694, Ile720, Lys721 and Met742. All these residues are located near the gatekeeper residue Thr766 (Thr790 in alternative numbering in EGFR), in which its location controls the access of an inhibitor to the hydrophobic pocket of the ATP-competitive site. Moreover, there are many acidic residues (Asp831, Asp 776 and Glu780) located on the phosphate binding region along the sugar pocket. Notably, unlike quinazoline compounds, our analyses also showed that these hit compounds are oriented, to some extent, away from the hinge region (Gln767-Met769) and the key hydrogen bonds, NH---N and CO---HC types, which are the most common kinase - inhibitor interaction, are rarely observed between that region and the compounds.

According to the differential GOLD scores as shown in Table 1, hydrophobic (lipophilic) scores were shown to be higher than the other two (hydrogen bond scores and scores for clash penalty) in all eight compound docks, suggesting that the main contribution of the binding is hydrophobic interaction. Non or low hydrogen-bond scores also indicated that strong hydrogen bond types may not be involved.

Compound B attaches to the adenine binding and the hydrophobic region I and II, with hydrophobic interaction force. The phenyl ring of compound B, located in hydrophobic I, form a π-π stacking interaction with Phe699 located in the glycine-rich nucleotide phosphate binding loop (P-loop). Furthermore, the π-cation interaction between the Nζ of Lys721 and Phenyl group of this compound was investigated. We also found the weak hydrogen bond; NH---O type at residue Met769 and O-atom of this compound (D-H distance > 3.0 Å). The binding modes of compound G are a combination of hydrophobic interaction and sidechain hydrogen bond interactions. This compound is mainly located in hydrophobic region I and II where one of the O-atom of the dioxoanthracene group forms a hydrogen bond to the H-atom of the Nζ from Lys721 in the minor pocket next to the ATP-binding region with in distance ≈ 1.9 Å. While the O-atom of the sulfophenyl group forms a hydrogen bond to the Cys773 at the H-atom from Sγ and the backbone-N in the sugar pocket and the H-atom of hydroxyl in the sulfophenyl group form hydrogen bond to the Oδ1 of Asp776 (the receptor exposure residue). The 6-chloro-2-methoxyacridin-9-yl group of compound H oriented the Cl-atom into the adenine pocket and the hydrophobic region I. The pose of this group is located similar to the quinazoline ring of erlotinib, while the H-atom of the phenyl group of this compound forms a hydrogen bond to the backbone carbonyl oxygen of the residue Arg817 located in the catalytic loop. Hydrophobic interactions were mainly present in compound I to L. The Nζ Lys721 residue establishes a cation-π interaction with the phenyl group to compound I. Compound J forms a weak hydrogen bond between the backbone-N of Met769 and the carbonyl oxygen of the dioxoanthracene-ring of the ligand. Compound K interacts with the kinase domain with hydrophobic force (π−π interaction) and weak hydrogen bond between the O-atom and the H-atom at Sγ position and the H-atom of backbone-N of Cys773. Similar to compound I with the hydrophobic force, there is the cation-π interaction from Nζ of Lys721 to the benzene ring of the compound L. In Table 1, compound M, which shows the lowest estimated binding free energy (ΔG = -44.57 kJ/mol), represents the cation-π interaction between the N-atom of the imine group and the Phe699, which in the glycine-rich nucleotide phosphate binding loop. Moreover, there is the π-π interaction between this residue with one of the phenazine ring and the Nζ of the Lys721 forms the cation-π interaction to the phenyl ring of the other core group. This compound orients one unit of 8-iodo-10-phenyl-5H-phenazin in the ATP-binding pocket and other unit in the minor pocket (Figure 4C). Furthermore, the results show that there is interaction between the I-atom of this compound and backbone-N of residue Met769, so called the induced-dipole interaction. Superimposition of our hit compounds with erlotinib also indicates the almost unique interaction between the kinase and a functional group of such compounds, and no similar pattern of the binding has been observed.

Consequently, our eight candidate compounds have the potential to be considered as new EGFR inhibitors. A combined computational approach, molecular dynamics simulation and calculation of binding free energy, is needed to investigate the detail interaction between the compounds and the protein. In addition, experimental procedures including protein crystallography, testing on the inhibitory activities against EGFR, other biological assays and animal studies will be required for the pathways on the drug development. Since the candidate compounds were tested for growth inhibition of cancer cell lines derived from patients with lung, central nervous system (CNS), melanoma, ovarian, renal, prostate and breast cancer (supplementary data S1). Thus, it would be interesting to investigate these compounds being the potential against other types of cancer.

## 3. Experimental

### 3.1. Preparation of the protein structure

The X-ray crystal structure of EGFR-TK complexed with the anti-cancer drug erlotinib was obtained from the Protein Data Bank (accession number 1M17) with 2.6 Å resolution [17]. The atomic coordinates of the protein was separated and its geometry optimized with Tripos SYBYL 7.3 (Tripos Associates, St. Louis, MO, USA) [41]. For docking with AutoDock, polar hydrogen atoms, Kollman united charges, and solvent parameters were applied to the protein by using pmol2q script [42]. This script converts the pdb file format of the protein template to the pdbqs file format compatible with the AutoDock program version 3.0.5 [43]. For docking with GOLD, hydrogen atoms were added into the protein structure using *“protonation and tautomers”* function in the configuration option of the *Gold Setup* window under GOLD package [44].

### 3.2. Preparation of the ligand structure

The coordinate files of the NCI diversity dataset were taken from the Office of the Associated Director of the Developmental Therapeutics Program, Division of Cancer Treatment and Diagnosis, National Cancer Institute in sdf MDL MOL format, more information is available at NCI/DTP Open Chemical Repository [45]. The dataset contains 1,990 chemical structures. All atomic coordinates were converted to SYBYL MOL2 format using OpenBabelGUI © 2006 (developed by Chris Morley; [46]. The single MOL2 file containing a number of molecules were split into individual single-molecule files using splitmol2 program [47]. The ligand pdbq files compatible with the AutoDock program version 3.0.5 were prepared from the MOL2 files by using prepare_ligand.py script (Scripps Research Institute). The parameters for rotational bonds and Gasteiger charges, of each ligand were assigned.

### 3.3. Molecular docking and post-docking analysis

#### 3.3.1. AutoDock 3.0.5

High-throughput docking-based virtual screening was performed by using AutoDock program version 3.0.5. The rotational bonds of the ligands were treated as flexible while those of the protein were kept rigid. Grid boxes were fixed around the ATP-binding site using erlotinib as the grid box center. The box size was set to 70, 60 and 60 Å^3^ (x, y and z, respectively) and the grid spacing to 0.375Å. The grid maps for twelve atom types (C: both aliphatic and aromatic carbons, N, O, S, H, P, Fe and halides: Br, Cl, F and I) were calculated. Genetic Algorithm (GA) was used for searching and the population size was set to 150. The scoring functions for the interaction were calculated using ChemGauss, ChemScore, Piecewise Linear Potential (PLP), Screenscore and Shapegauss from FRED program [48]. The compounds with ranking of at least four out of five were selected for docking evaluation.

#### 3.3.2. GOLD 4.0.1

Automatic GA parameter setting was used in all of the GOLD docking calculations. A hundred percent search efficiency was applied, with a minimum of 10,000 and a maximum of 125,000 operations per ligand. The binding site was defined to include all amino acid residues within a 7 Å radii from the center of erlotinib; all of the water molecules were removed. The kinase scoring function (KCS), modified from the ChemScore fitness function, and was applied in all the docking calculations. This scoring function includes the contributions of the weak CH∙∙∙O hydrogen bond which are mostly found in kinase proteins. The diverse solutions are generated and the early termination is allowed when a number of similar docking solutions for the particular ligand are obtained.

#### 3.3.3. Structural analysis and visualization

Protein-ligand interaction was analyzed and visualized by Discovery Studio 2.5 (Accelrys Inc., San Diego, CA, USA), MOE (Chemical Computing Group Inc., Montreal, Quebec, Canada) [49], VIDA 3.0.0 [50] and PyMol 0.99 programs [51].

## 4. Conclusions

In this work, we have searched for novel anti-EGFR inhibitors through molecular docking-based virtual screening of 1,990 chemical compounds form NCI diversity set. We have also shown that it is unnecessary for receptor-based virtual screening to include water molecules into the EGFR-binding pocket since all EGFR-TK structures contain no conserved position for water molecules. In fact, our self-docking study of erlotinib to EGFR-TK without any water molecules led to correct results. Binding mode analyses revealed that the interaction between our eight candidate compounds with high rank scoring and the EGFR-TK is different from that of anilinoquinazoline inhibitors. We found the induced-dipole, cation-π and π-π interactions and other weak hydrogen bonds around the binding site rather than the common kinase-inhibitor (NH---N and CO---HC) interaction the hinge residues and the hit molecules. However, the amino acid residues located in the hydrophobic pocket (Lys721, Met742, Leu764 and Ile765) have been shown to interact with aromatic ring of almost all compounds. This pocket was found to accommodate the aniline moiety of the known kinase inhibitors. Moreover, high scores of hydrophobic property calculated from the GOLD program suggest that the main contribution of the EGFR-TK with these candidate compounds is hydrophobic interaction. These findings demonstrated that these compounds could be developed as novel lead compounds for designing of anti-cancer drugs. Further experiments would be required for investigating the detailed interaction and *in vitro* testing their inhibitory activity against EGFR.

## Supplementary Materials

Supplementary File 1
