# Supplementary material for: Receptor-Based Virtual Screening of EGFR Kinase Inhibitors from the NCI Diversity Database
Source: Molecules. 2010 Jun 4;15(6):4041–54. doi: 10.3390/molecules15064041 (PMC6264413; doi:10.3390/molecules15064041)
Supplement: Supplementary File 1 [file molecules-15-04041-s001.pdf]

Supplementary data

S1. The explanation for the Mean Graph of GI_{50}: the mean GI_{50} graph demonstrated the selective nature of growth inhibition of human derived cancer cell lines. The positive and negative values means resistance and sensitivity of a particular cell line, respectively.

Erlotinib

GI_{50} Mean Graph for Compound 718781

NCI Cancer Screen Current Data, May 2009

Average GI_{50} over all cell lines is 5.51E-6

Cell Panel	Cell Line	Log GI_{50}
Leukemia	CCRF-CEM	-4.8
	HL-60(TB)	-5.3
	K-562	-4.8
	MOLT-4	-5.3
	RPMI-8226	-5.3
	SR	-5.2
Non-Small Cell Lung	A549/ATCC	-5.1
	EKVX	-7.3
	HOP-62	-4.9
	HOP-92	-5.2
	NCI-H226	-4.6
	NCI-H23	-4.7
	NCI-H322M	-7.3
	NCI-H460	-5.3
	NCI-H522	-6.0
Colon	COLO 205	-4.5
	HCC-2998	-4.1
	HCT-116	-5.2
	HCT-15	-5.5
	HT29	-4.3
	KM12	-4.2
	SW-620	-4.3
Central Nervous System	SF-268	-4.7
	SF-295	-4.8
	SF-539	-4.9
	SNB-19	-5.4
	SNB-75	-4.9
	U251	-4.7
Melanoma	LOX IMVI	-5.3
	MALME-3M	-5.3
	M14	-5.2
	MDA-MB-435	-4.8
	SK-MEL-2	-4.9
	SK-MEL-28	-4.5
	SK-MEL-5	-4.8
	UACC-257	-4.0
	UACC-62	-5.9
Ovarian	IGROV1	-6.6
	OVCAR-3	-5.5
	OVCAR-4	-4.7
	OVCAR-5	-4.7
	OVCAR-8	-5.1
	NCI/ADR-RES	-5.2
	SK-OV-3	-6.4
Renal	786-0	-5.3
	A498	-5.8
	ACHN	-6.8
	CAKI-1	-7.0
	RXF 393	-5.2
	SN12C	-6.2
	TK-10	-7.0
	UO-31	-5.7
Prostate	PC-3	-4.3
	DU-145	-5.8
Breast	MCF7	-4.0
	MDA-MB-231/ATCC	-5.7
	HS 578T	-5.2
	MDA-N	-4.4
	BT-549	-4.6
	T-47D	-5.5
	MDA-MB-468	-6.7

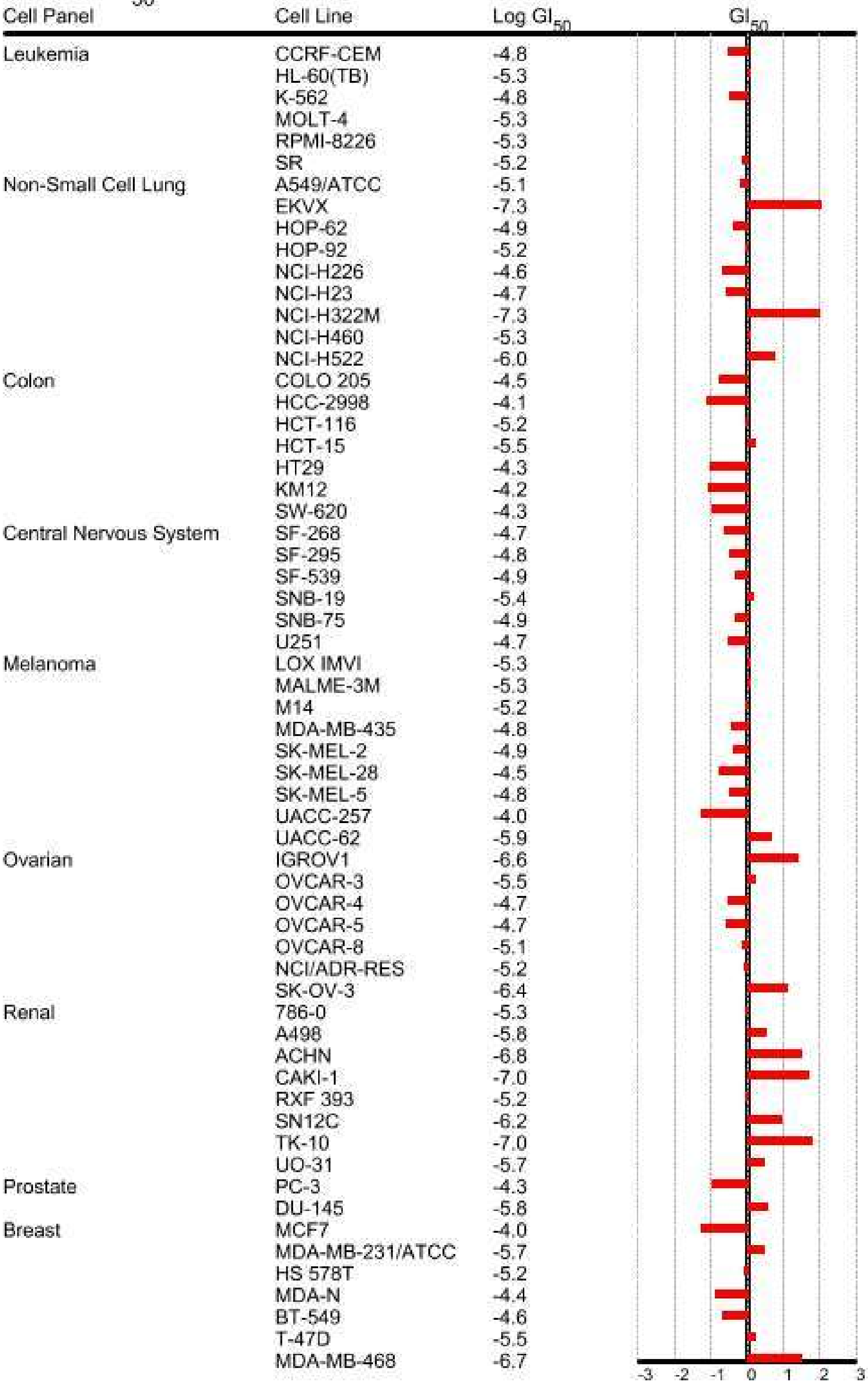

Gefitinib

GI_{50} Mean Graph for Compound 715055

NCI Cancer Screen Current Data, October 2009

Average GI_{50} over all cell lines is 3.24E-6

Cell Panel	Cell Line	Log GI_{50}
Leukemia	CCRF-CEM	-5.3
	HL-60(TB)	-5.3
	K-562	-5.6
	MOLT-4	-5.4
	RPMI-8226	-5.8
	SR	-5.5
Non-Small Cell Lung	A549/ATCC	-5.1
	EKVX	-7.3
	HOP-62	-5.0
	HOP-92	-5.1
	NCI-H226	-4.8
	NCI-H23	-4.8
	NCI-H322M	-7.1
	NCI-H460	-5.2
	NCI-H522	-5.2
Colon	COLO 205	-5.2
	HCC-2998	-5.0
	HCT-116	-5.1
	HCT-15	-5.3
	HT29	-5.4
	KM12	-5.1
	SW-620	-5.1
Central Nervous System	SF-268	-5.1
	SF-295	-5.7
	SF-539	-5.0
	SNB-19	-4.9
	SNB-75	-5.2
	U251	-5.0
Melanoma	LOX IMVI	-5.1
	MALME-3M	-5.5
	M14	-5.3
	MDA-MB-435	-5.5
	SK-MEL-2	-4.9
	SK-MEL-28	-6.5
	SK-MEL-5	-5.4
	UACC-257	-5.2
	UACC-62	-5.3
Ovarian	IGROV1	-6.7
	OVCAR-3	-5.3
	OVCAR-4	-5.1
	OVCAR-5	-5.0
	OVCAR-8	-5.0
	NCI/ADR-RES	-4.9
	SK-OV-3	-6.2
Renal	786-0	-5.1
	A498	-6.4
	ACHN	-6.7
	CAKI-1	-6.8
	RXF 393	-5.3
	SN12C	-5.2
	TK-10	-7.0
	UO-31	-5.9
Prostate	PC-3	-6.1
	DU-145	-5.6
Breast	MCF7	-5.0
	MDA-MB-231/ATCC	-4.9
	HS 578T	-5.0
	BT-549	-5.1
	T-47D	-5.2
	MDA-MB-468	-8.0

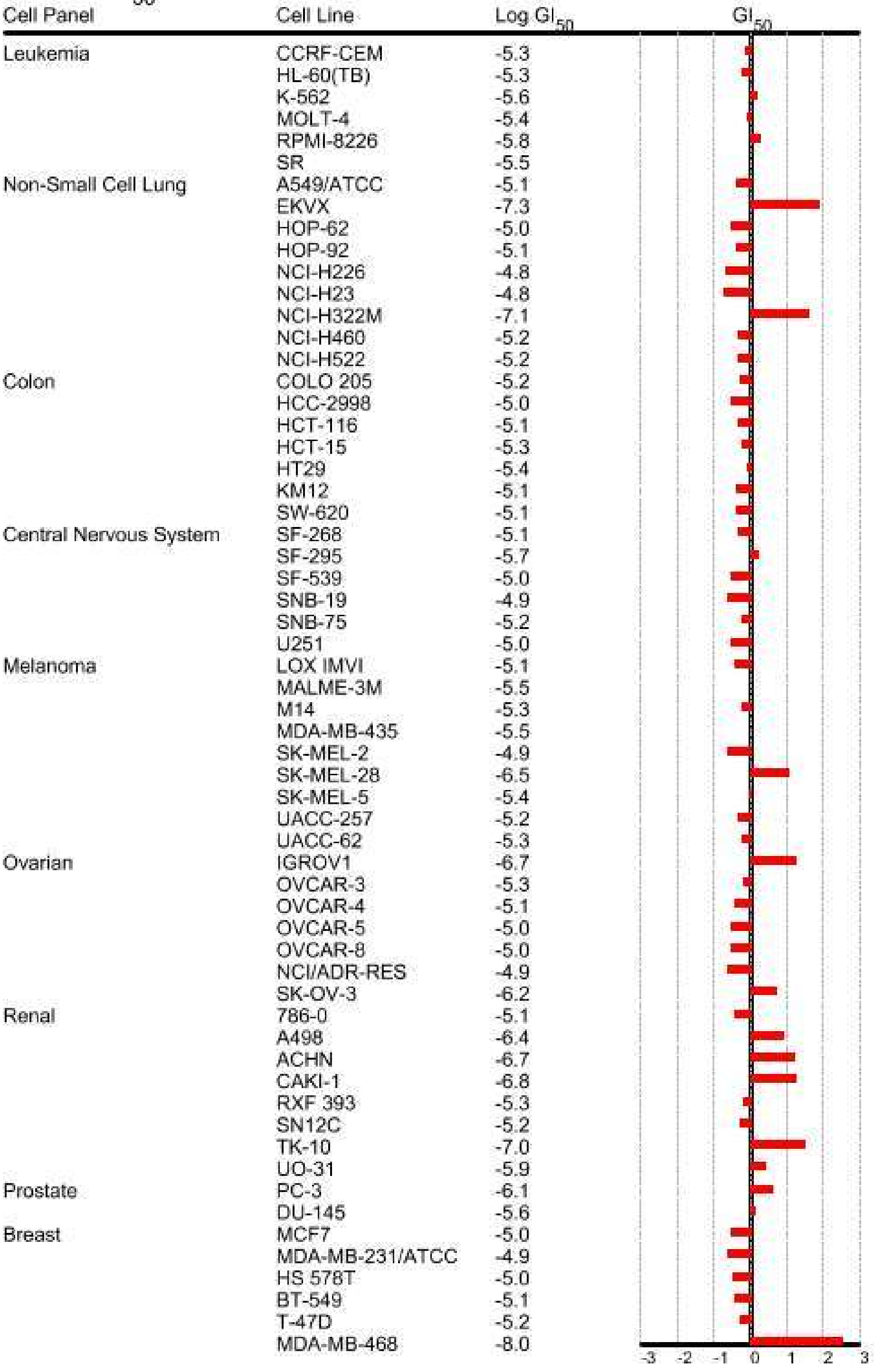

Compound B

GI_{50} Mean Graph for Compound 48283

NCI Cancer Screen Current Data, May 2009

Average GI_{50} over all cell lines is 9.76E-5

Cell Panel	Cell Line	Log GI_{50}
Leukemia	CCRF-CEM	-4.0
	K-562	-4.0
	MOLT-4	-4.0
	RPMI-8226	-4.0
	SR	-4.0
Non-Small Cell Lung	A549/ATCC	-4.0
	EKVX	-4.0
	HOP-62	-4.0
	HOP-92	-4.0
	NCI-H23	-4.0
	NCI-H322M	-4.0
	NCI-H460	-4.0
	NCI-H522	-4.6
Colon	COLO 205	-4.0
	HCT-116	-4.0
	HCT-15	-4.0
	HT29	-4.0
	KM12	-4.0
	SW-620	-4.0
Central Nervous System	SF-268	-4.0
	SF-295	-4.0
	SF-539	-4.0
	SNB-19	-4.0
	SNB-75	-4.0
	U251	-4.0
Melanoma	LOX IMVI	-4.0
	MALME-3M	-4.0
	M14	-4.0
	MDA-MB-435	-4.0
	SK-MEL-2	-4.0
	SK-MEL-28	-4.0
	SK-MEL-5	-4.0
	UACC-257	-4.0
	UACC-62	-4.0
Ovarian	IGROV1	-4.0
	OVCAR-3	-4.0
	OVCAR-4	-4.0
	OVCAR-5	-4.0
	OVCAR-8	-4.0
	NCI/ADR-RES	-4.0
	SK-OV-3	-4.0
Renal	786-0	-4.0
	A498	-4.0
	ACHN	-4.0
	CAKI-1	-4.0
	RXF 393	-4.0
	SN12C	-4.0
	TK-10	-4.0
	UO-31	-4.0
Prostate	PC-3	-4.0
	DU-145	-4.0
Breast	MCF7	-4.0
	MDA-MB-231/ATCC	-4.0
	HS 578T	-4.0
	MDA-N	-4.0
	BT-549	-4.0
	T-47D	-4.0

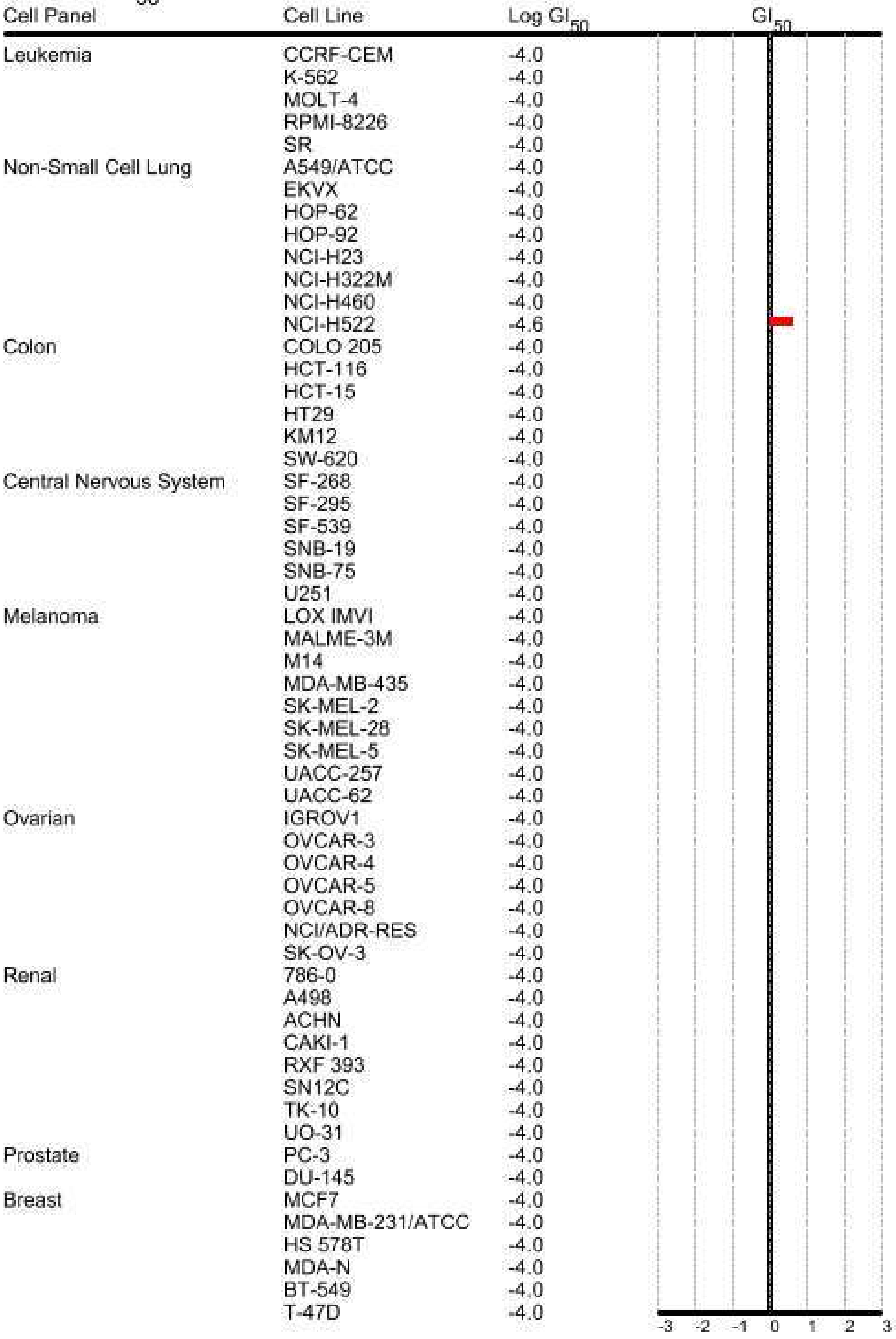

Compound G

GI_{50} Mean Graph for Compound 125910

NCI Cancer Screen Current Data, May 2009

Average GI_{50} over all cell lines is 9.86E-5

Cell Panel	Cell Line	Log GI_{50}
Leukemia	CCRF-CEM	-4.0
	HL-60(TB)	-4.0
	K-562	-4.0
	MOLT-4	-4.4
	RPMI-8226	-4.0
	SR	-4.0
Non-Small Cell Lung	A549/ATCC	-4.0
	EKVX	-4.0
	HOP-62	-4.0
	HOP-92	-4.0
	NCI-H226	-4.0
	NCI-H23	-4.0
	NCI-H322M	-4.0
	NCI-H460	-4.0
	NCI-H522	-4.0
Colon	COLO 205	-4.0
	HCC-2998	-4.0
	HCT-116	-4.0
	HCT-15	-4.0
	HT29	-4.0
	KM12	-4.0
	SW-620	-4.0
Central Nervous System	SF-268	-4.0
	SF-295	-4.0
	SF-539	-4.0
	SNB-19	-4.0
	SNB-75	-4.0
	U251	-4.0
Melanoma	LOX IMVI	-4.0
	MALME-3M	-4.0
	M14	-4.0
	MDA-MB-435	-4.0
	SK-MEL-2	-4.0
	SK-MEL-28	-4.0
	SK-MEL-5	-4.0
	UACC-257	-4.0
	UACC-62	-4.0
Ovarian	IGROV1	-4.0
	OVCAR-3	-4.0
	OVCAR-4	-4.0
	OVCAR-5	-4.0
	OVCAR-8	-4.0
	NCI/ADR-RES	-4.0
	SK-OV-3	-4.0
Renal	786-0	-4.0
	A498	-4.0
	ACHN	-4.0
	CAKI-1	-4.0
	RXF 393	-4.0
	SN12C	-4.0
	TK-10	-4.0
	UO-31	-4.0
Prostate	PC-3	-4.0
	DU-145	-4.0
Breast	MCF7	-4.0
	MDA-MB-231/ATCC	-4.0
	HS 578T	-4.0
	MDA-N	-4.0
	BT-549	-4.0
	T-47D	-4.0

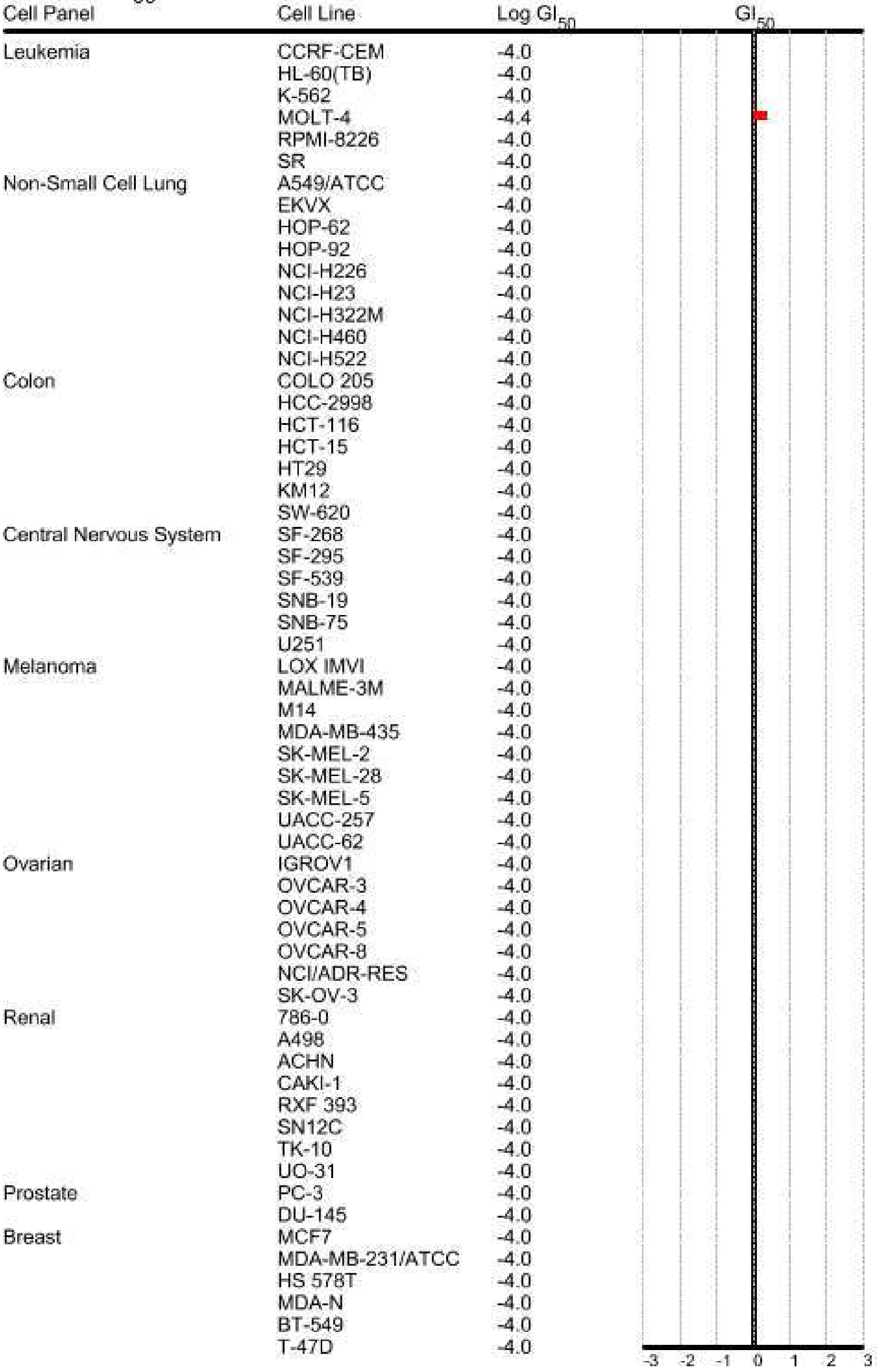

Compound H

GI$_{50}$ Mean Graph for Compound 130813

NCI Cancer Screen Current Data, May 2009

Average GI$_{50}$ over all cell lines is 1.94E-6

Cell Panel	Cell Line	Log GI$_{50}$
Leukemia	CCRF-CEM	-6.6
	HL-60(TB)	-5.7
	K-562	-5.8
	MOLT-4	-5.7
	RPMI-8226	-5.7
	SR	-6.2
Non-Small Cell Lung	A549/ATCC	-5.7
	EKVX	-5.8
	HOP-62	-5.7
	HOP-92	-5.8
	NCI-H226	-4.7
	NCI-H23	-5.7
	NCI-H322M	-5.8
	NCI-H460	-5.8
	NCI-H522	-5.8
Colon	COLO 205	-5.8
	HCC-2998	-5.9
	HCT-116	-5.8
	HCT-15	-5.6
	HT29	-5.8
	KM12	-5.7
	SW-620	-5.7
Central Nervous System	SF-268	-5.6
	SF-295	-5.7
	SF-539	-5.7
	SNB-19	-5.6
	SNB-75	-5.8
	U251	-5.8
Melanoma	LOX IMVI	-5.8
	MALME-3M	-5.7
	M14	-5.7
	MDA-MB-435	-5.8
	SK-MEL-2	-5.7
	SK-MEL-28	-5.7
	SK-MEL-5	-5.8
	UACC-257	-5.7
	UACC-62	-5.8
Ovarian	IGROV1	-5.8
	OVCAR-3	-5.7
	OVCAR-4	-5.8
	OVCAR-5	-5.6
	OVCAR-8	-5.7
	NCI/ADR-RES	-5.7
	SK-OV-3	-5.2
Renal	786-0	-5.8
	A498	-5.3
	ACHN	-5.8
	CAKI-1	-5.7
	RXF 393	-5.8
	SN12C	-5.7
	TK-10	-5.7
	UO-31	-5.7
Prostate	PC-3	-5.8
	DU-145	-5.7
Breast	MCF7	-5.8
	MDA-MB-231/ATCC	-5.8
	HS 578T	-5.7
	MDA-N	-5.8
	BT-549	-4.7
	T-47D	-5.7

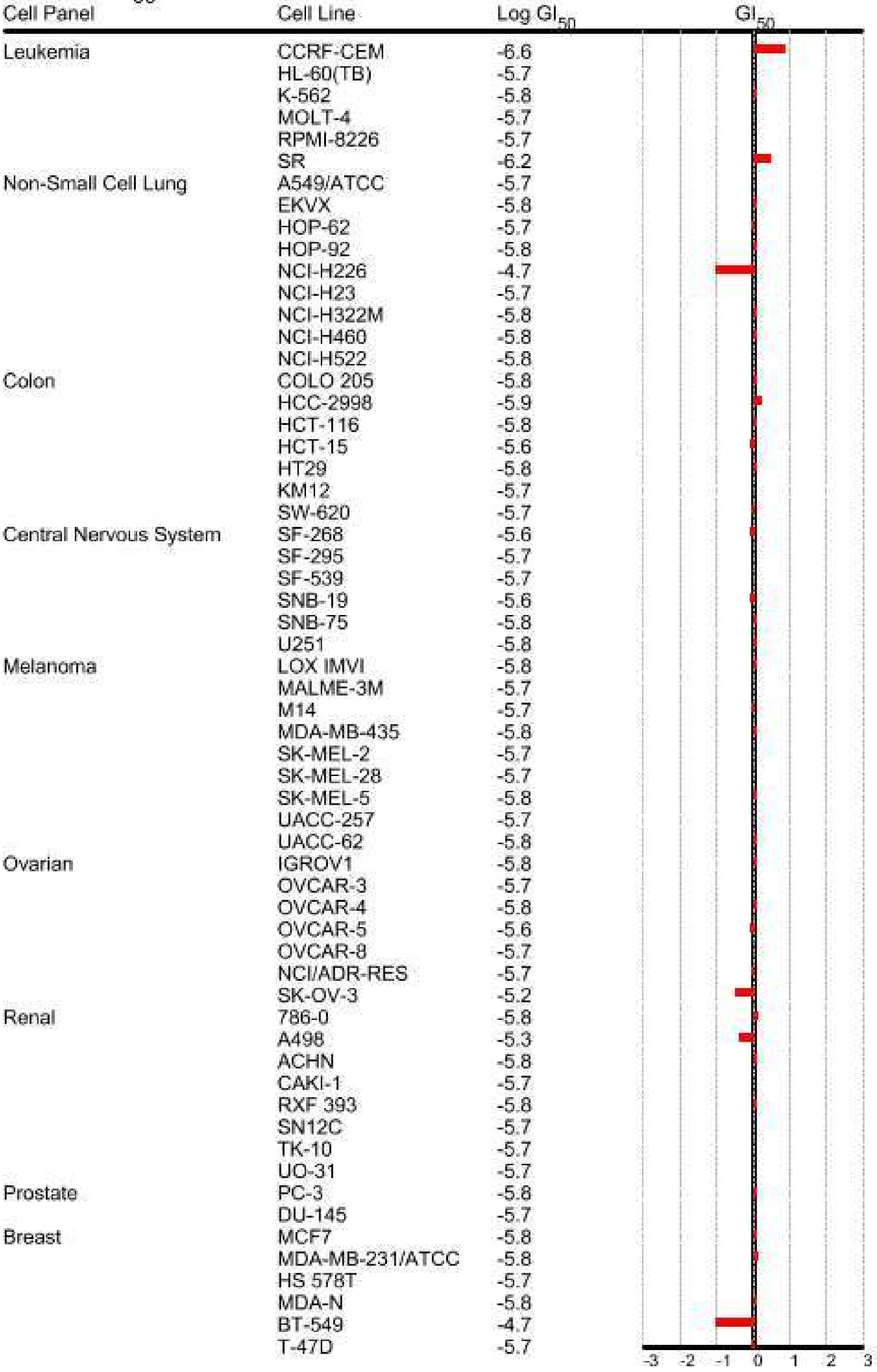

Compound I

GI_{50} Mean Graph for Compound 135371

NCI Cancer Screen Current Data, May 2009

Average GI_{50} over all cell lines is 3.95E-5

Cell Panel	Cell Line	Log GI_{50}
Leukemia	CCRF-CEM	-5.6
	HL-60(TB)	-5.5
	K-562	-5.3
	MOLT-4	-5.3
	RPMI-8226	-5.3
	SR	-5.2
Non-Small Cell Lung	A549/ATCC	-5.1
	EKVX	-4.0
	HOP-62	-4.0
	HOP-92	-5.1
	NCI-H226	-4.0
	NCI-H23	-4.0
	NCI-H322M	-4.0
	NCI-H460	-4.8
	NCI-H522	-5.0
Colon	COLO 205	-4.8
	HCT-116	-5.2
	HCT-15	-4.3
	HT29	-5.0
	KM12	-4.6
	SW-620	-4.5
Central Nervous System	SF-268	-4.0
	SF-295	-4.0
	SF-539	-4.0
	SNB-19	-4.0
	SNB-75	-4.0
	U251	-4.1
Melanoma	LOX IMVI	-4.0
	MALME-3M	-4.0
	M14	-4.0
	MDA-MB-435	-4.5
	SK-MEL-2	-4.0
	SK-MEL-28	-5.0
	SK-MEL-5	-4.3
	UACC-257	-4.0
	UACC-62	-4.0
Ovarian	IGROV1	-4.0
	OVCAR-3	-4.8
	OVCAR-4	-5.1
	OVCAR-5	-4.0
	OVCAR-8	-4.0
	NCI/ADR-RES	-4.6
	SK-OV-3	-4.0
Renal	786-0	-4.0
	A498	-4.0
	ACHN	-4.0
	CAKI-1	-4.0
	RXF 393	-4.0
	SN12C	-4.4
	TK-10	-4.0
	UO-31	-4.3
Prostate	PC-3	-4.5
	DU-145	-4.0
Breast	MCF7	-4.5
	MDA-MB-231/ATCC	-4.0
	HS 578T	-4.0
	MDA-N	-4.0
	BT-549	-4.0
	T-47D	-5.0

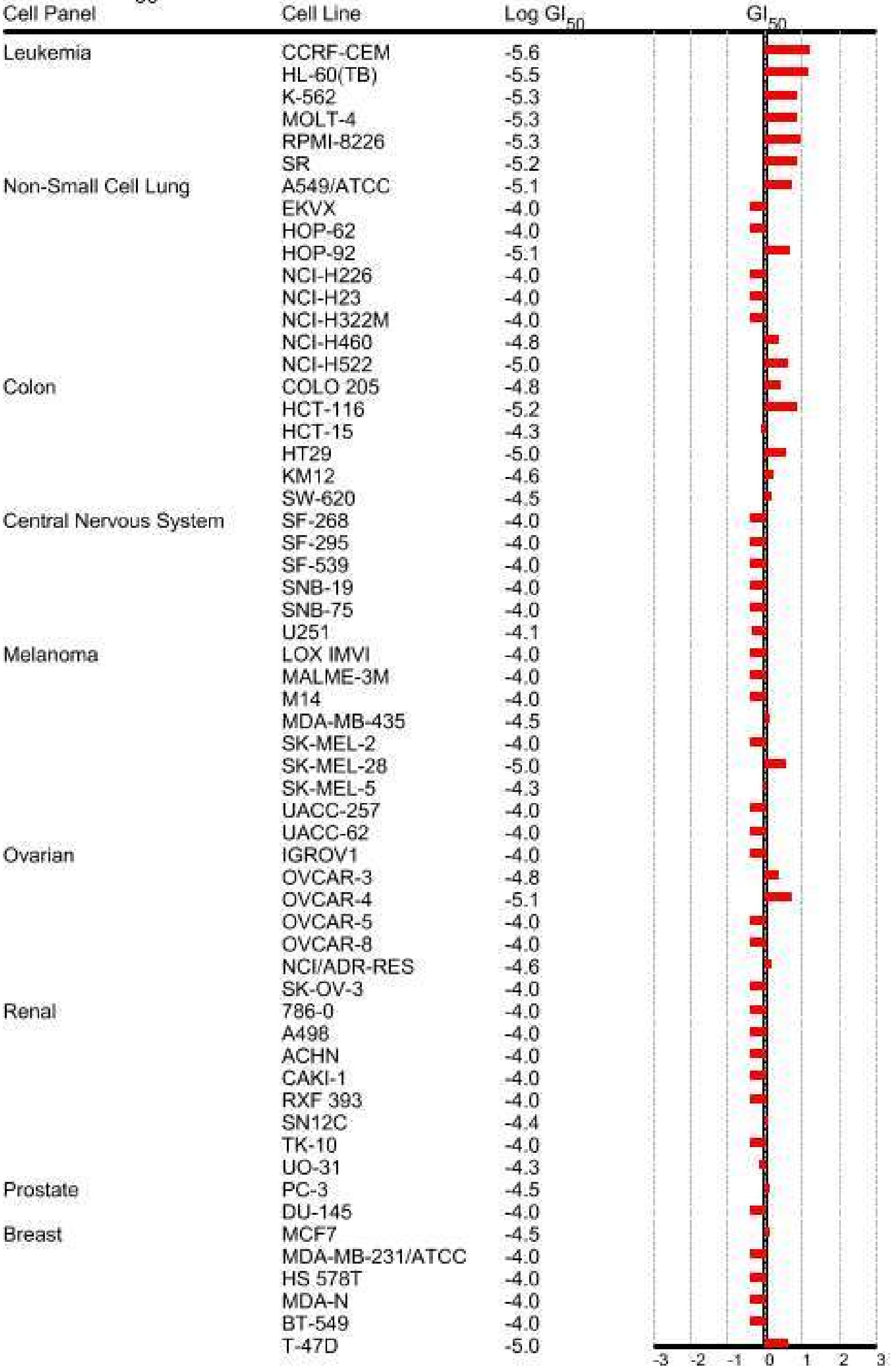

Compound J

GI_{50} Mean Graph for Compound 299137

NCI Cancer Screen Current Data, May 2009

Average GI_{50} over all cell lines is 7.03E-5

Cell Panel	Cell Line	Log GI_{50}
Leukemia	K-562	-4.0
	MOLT-4	-4.0
	RPMI-8226	-4.0
	SR	-4.0
Non-Small Cell Lung	A549/ATCC	-4.0
	EKVX	-4.0
	HOP-62	-4.8
	HOP-92	-4.3
	NCI-H226	-4.0
	NCI-H23	-4.5
	NCI-H322M	-4.0
	NCI-H460	-4.0
	NCI-H522	-4.0
Colon	COLO 205	-4.0
	HCC-2998	-4.0
	HCT-116	-4.0
	HCT-15	-4.0
	HT29	-4.0
	KM12	-4.0
	SW-620	-4.0
Central Nervous System	SF-268	-4.0
	SF-295	-4.6
	SF-539	-4.3
	SNB-19	-4.5
	SNB-75	-4.7
	U251	-4.5
Melanoma	MALME-3M	-4.0
	M14	-4.0
	MDA-MB-435	-4.0
	SK-MEL-2	-4.0
	SK-MEL-28	-4.0
	SK-MEL-5	-4.0
	UACC-257	-4.0
	UACC-62	-4.0
Ovarian	IGROV1	-4.0
	OVCAR-3	-4.4
	OVCAR-4	-4.0
	OVCAR-5	-4.0
	OVCAR-8	-4.0
	NCI/ADR-RES	-4.0
	SK-OV-3	-4.6
Renal	786-0	-4.6
	A498	-4.4
	ACHN	-4.0
	CAKI-1	-4.0
	RXF 393	-4.6
	SN12C	-4.0
	TK-10	-4.7
	UO-31	-4.0
Prostate	PC-3	-4.0
	DU-145	-4.0
Breast	MCF7	-4.0
	MDA-MB-231/ATCC	-4.5
	HS 578T	-4.2
	MDA-N	-4.0
	BT-549	-4.3
	T-47D	-4.0

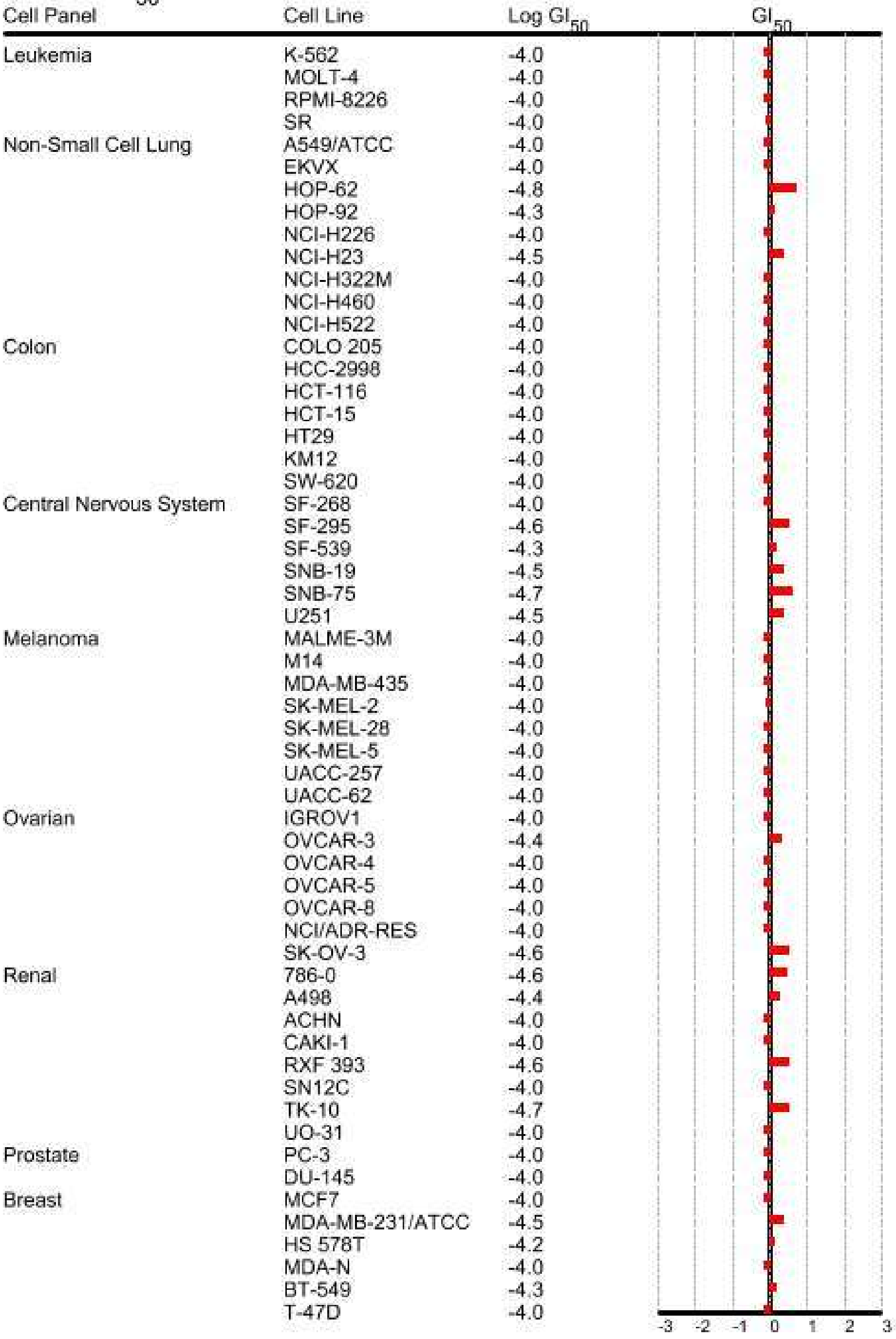

Compound K

GI$_{50}$ Mean Graph for Compound 306698
NCI Cancer Screen Current Data, May 2009
Average GI$_{50}$ over all cell lines is 3.25E-6

Cell Panel	Cell Line	Log GI$_{50}$
Leukemia	K-562	-5.4
	RPMI-8226	-5.7
Non-Small Cell Lung	A549/ATCC	-5.5
	EKVX	-5.6
	HOP-62	-5.4
	NCI-H226	-5.5
	NCI-H23	-5.6
	NCI-H322M	-5.6
	NCI-H460	-5.6
	NCI-H522	-5.5
Colon	COLO 205	-5.2
	HCC-2998	-5.6
	HCT-116	-5.6
	HCT-15	-5.5
	HT29	-5.5
	KM12	-5.5
	SW-620	-5.4
Central Nervous System	SF-268	-5.5
	SF-295	-5.4
	SF-539	-5.3
	SNB-19	-5.4
	SNB-75	-5.6
	U251	-5.5
Melanoma	MALME-3M	-5.5
	M14	-5.4
	MDA-MB-435	-5.5
	SK-MEL-2	-5.8
	SK-MEL-28	-5.2
	SK-MEL-5	-5.6
	UACC-257	-5.5
	UACC-62	-5.3
Ovarian	OVCAR-3	-5.4
	OVCAR-4	-5.4
	OVCAR-5	-5.4
	NCI/ADR-RES	-5.6
	SK-OV-3	-5.5
Renal	786-0	-5.4
	A498	-5.5
	ACHN	-5.4
	CAKI-1	-5.5
	RXF 393	-5.7
	SN12C	-5.5
	TK-10	-5.3
	UO-31	-5.4
Prostate	PC-3	-5.7
	DU-145	-5.5
Breast	MCF7	-5.5
	MDA-MB-231/ATCC	-5.6
	HS 578T	-5.5
	MDA-N	-5.5
	BT-549	-5.6
	T-47D	-5.5

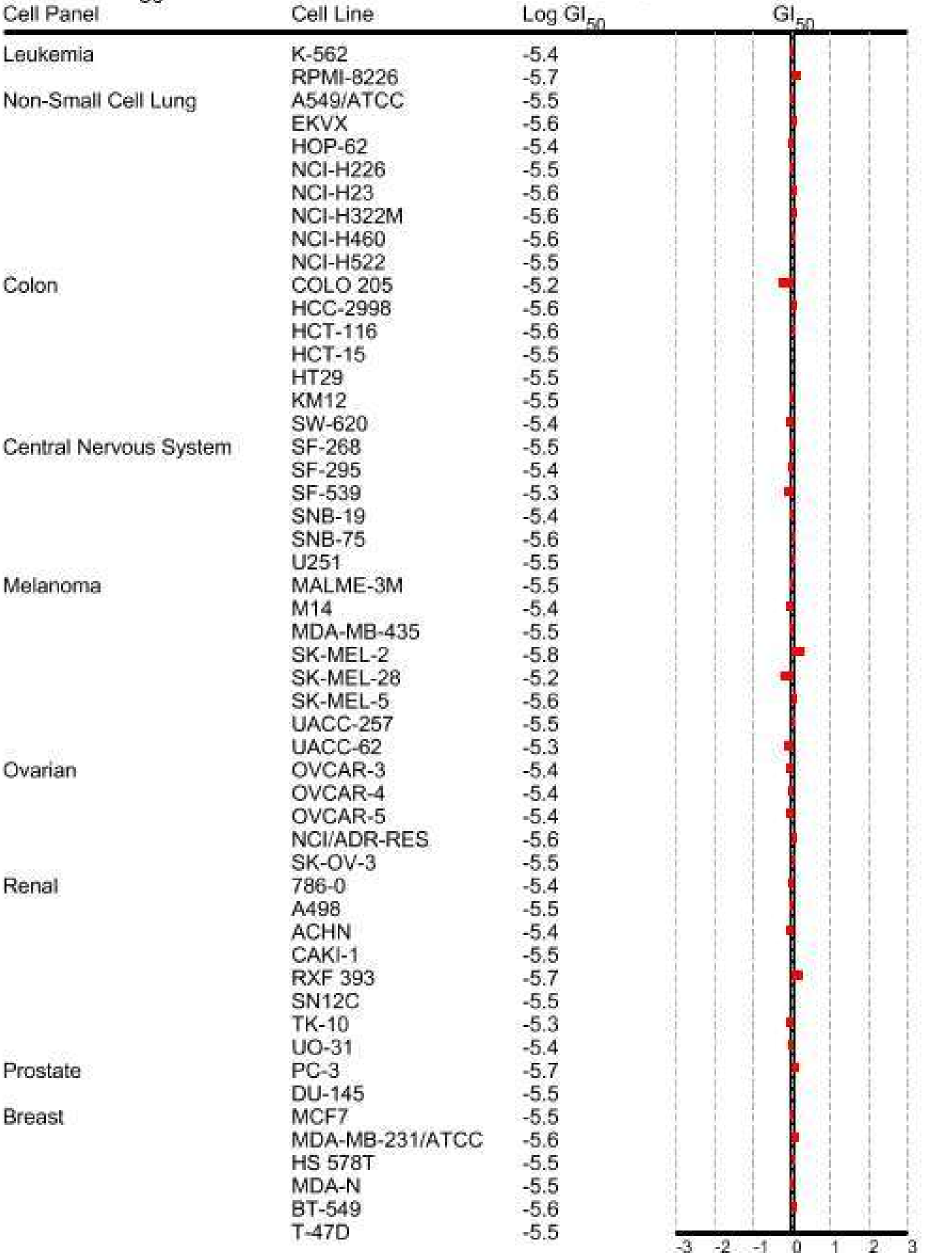

Compound L

GI$_{50}$ Mean Graph for Compound 351123

NCI Cancer Screen Current Data, May 2009

Average GI$_{50}$ over all cell lines is 9.02E-5

Cell Panel	Cell Line	Log GI$_{50}$
Leukemia	HL-60(TB)	-4.0
	K-562	-4.0
	MOLT-4	-4.6
	RPMI-8226	-4.0
	SR	-4.5
Non-Small Cell Lung	A549/ATCC	-4.1
	EKVX	-4.0
	HOP-62	-4.0
	HOP-92	-4.0
	NCI-H226	-4.0
	NCI-H23	-4.0
	NCI-H322M	-4.0
	NCI-H460	-4.4
	NCI-H522	-4.0
Colon	COLO 205	-4.0
	HCC-2998	-4.1
	HCT-116	-4.0
	HCT-15	-4.6
	HT29	-4.1
	KM12	-4.0
	SW-620	-4.0
Central Nervous System	SF-268	-4.0
	SF-295	-4.0
	SF-539	-4.0
	SNB-19	-4.0
	SNB-75	-4.0
	U251	-4.0
Melanoma	LOX IMVI	-4.0
	M14	-4.0
	MDA-MB-435	-4.0
	SK-MEL-2	-4.0
	SK-MEL-28	-4.0
	SK-MEL-5	-4.2
	UACC-257	-4.0
	UACC-62	-4.0
Ovarian	IGROV1	-4.0
	OVCAR-3	-4.0
	OVCAR-4	-4.0
	OVCAR-5	-4.0
	OVCAR-8	-4.0
	NCI/ADR-RES	-4.0
	SK-OV-3	-4.0
Renal	786-0	-4.0
	A498	-4.0
	ACHN	-4.0
	CAKI-1	-4.0
	RXF 393	-4.0
	SN12C	-4.0
	TK-10	-4.0
	UO-31	-4.0
Prostate	PC-3	-4.0
	DU-145	-4.0
Breast	MCF7	-4.0
	MDA-MB-231/ATCC	-4.0
	HS 578T	-4.0
	MDA-N	-4.0
	BT-549	-4.0
	T-47D	-4.0

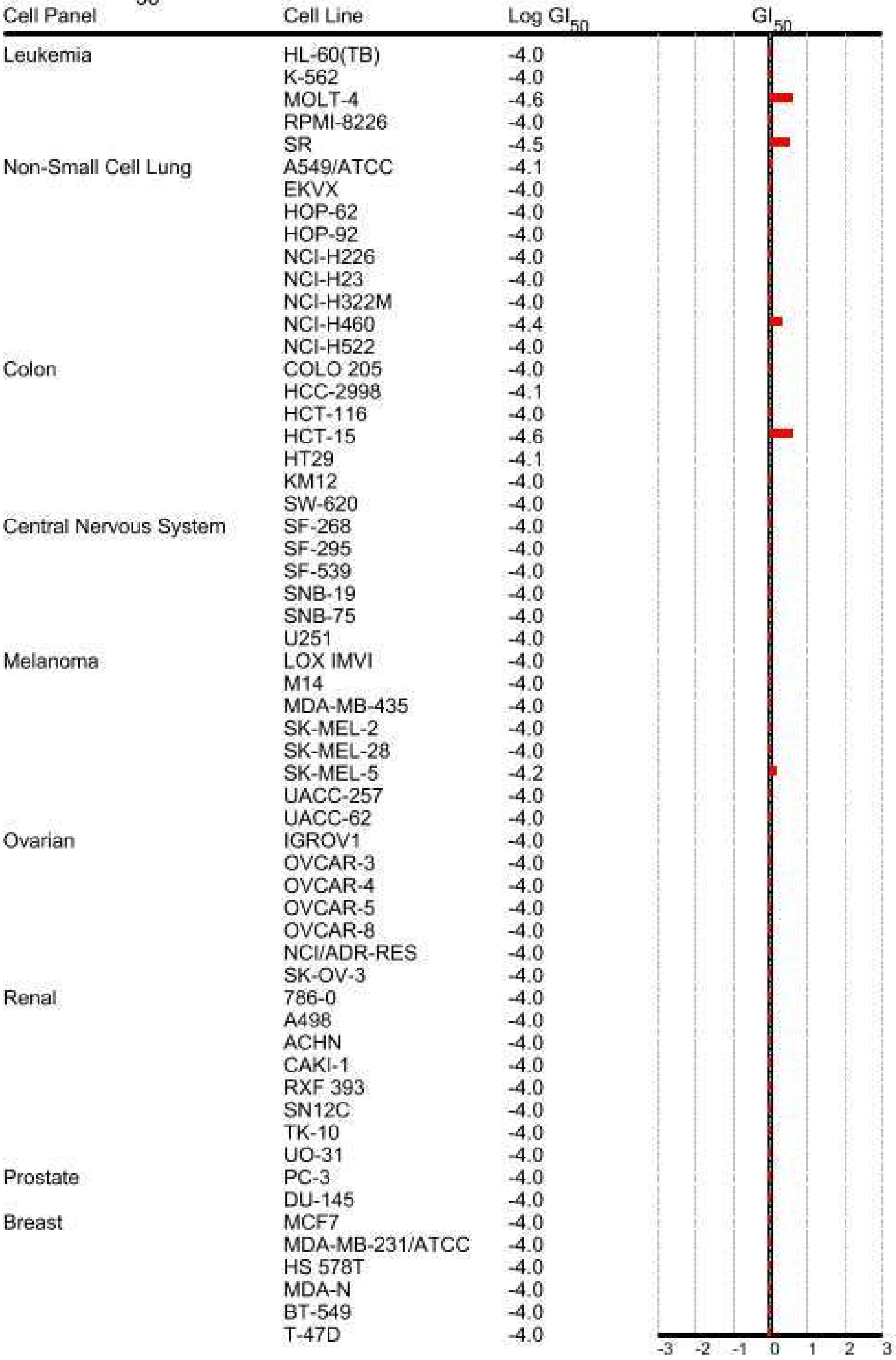

Compound M

GI_{50} Mean Graph for Compound 402959

NCI Cancer Screen Current Data, May 2009

Average GI_{50} over all cell lines is 5.5E-5

Cell Panel	Cell Line	Log GI_{50}
Leukemia	CCRF-CEM	-4.8
	HL-60(TB)	-4.7
	K-562	-4.5
	MOLT-4	-4.2
	RPMI-8226	-4.5
	SR	-4.5
Non-Small Cell Lung	A549/ATCC	-4.0
	EKVX	-4.5
	HOP-62	-4.4
	HOP-92	-4.0
	NCI-H226	-4.0
	NCI-H23	-4.4
	NCI-H322M	-4.0
	NCI-H460	-4.4
	NCI-H522	-4.6
Colon	COLO 205	-4.8
	HCC-2998	-4.2
	HCT-116	-4.5
	HCT-15	-4.0
	HT29	-4.8
	KM12	-4.4
	SW-620	-4.3
Central Nervous System	SF-268	-4.0
	SF-295	-4.0
	SF-539	-4.0
	SNB-19	-4.0
	SNB-75	-4.0
	U251	-4.3
Melanoma	MALME-3M	-4.4
	M14	-4.6
	MDA-MB-435	-4.5
	SK-MEL-2	-4.0
	SK-MEL-28	-4.0
	SK-MEL-5	-4.5
	UACC-257	-4.5
	UACC-62	-4.2
Ovarian	IGROV1	-4.4
	OVCAR-3	-5.5
	OVCAR-4	-4.0
	OVCAR-5	-4.0
	OVCAR-8	-4.0
	NCI/ADR-RES	-4.0
	SK-OV-3	-4.0
Renal	786-0	-4.0
	A498	-4.0
	ACHN	-4.0
	CAKI-1	-4.0
	RXF 393	-4.0
	SN12C	-4.0
	TK-10	-4.0
	UO-31	-4.0
Prostate	PC-3	-4.3
	DU-145	-4.0
Breast	MCF7	-4.6
	MDA-MB-231/ATCC	-4.6
	HS 578T	-4.0
	MDA-N	-4.4
	BT-549	-4.0
	T-47D	-4.2

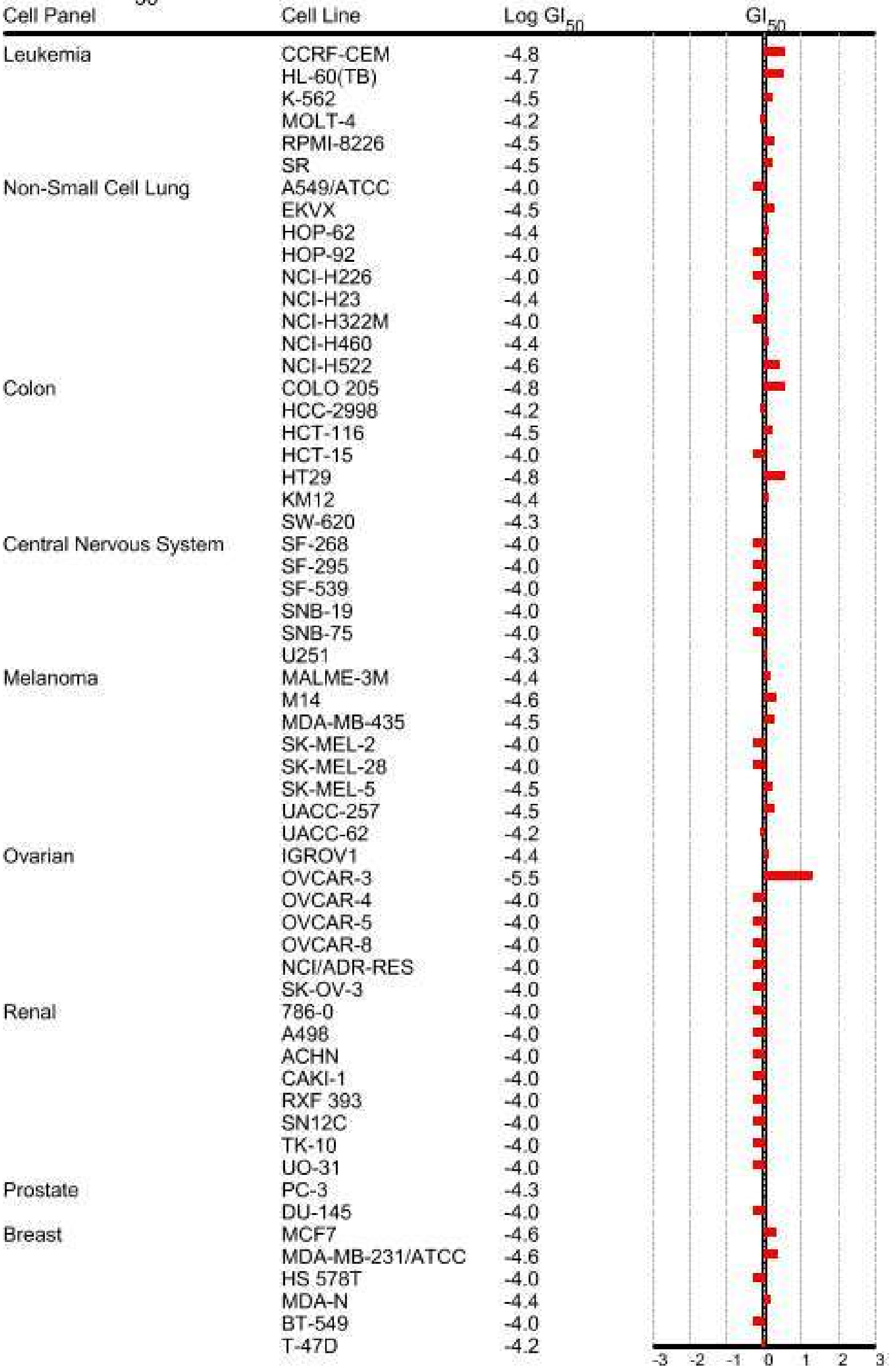

Sample Availability: Samples are available from the authors.

S2. Diagram showing the interaction between the EGFR-TK and the eight candidate compounds using Ligand Interactions module in MOE package.

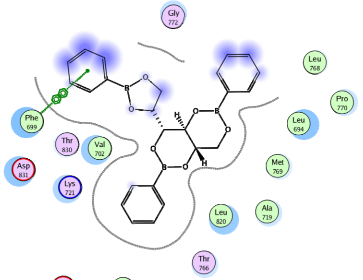

NSC 48283
Compound (B)

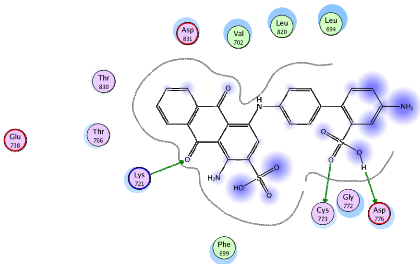

NSC 125910
Compound (G)

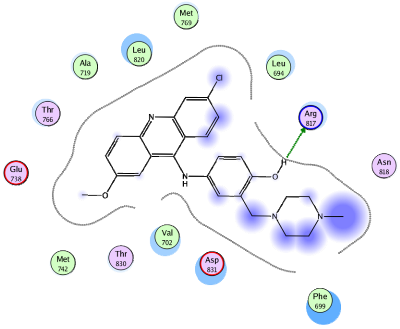

NSC 130813
Compound (H)

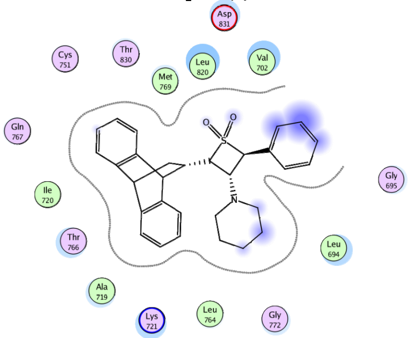

NSC 135371
Compound (I)

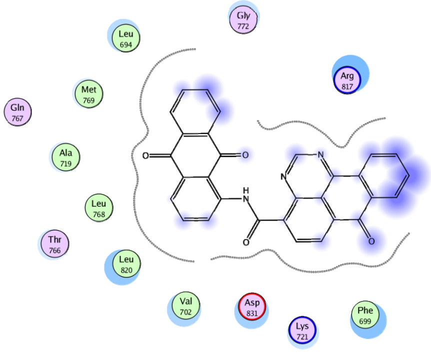

NSC 299137
Compound (J)

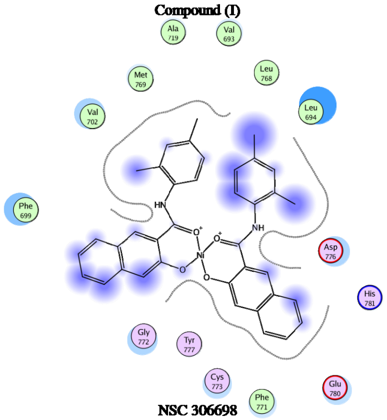

NSC 306698
Compound (K)

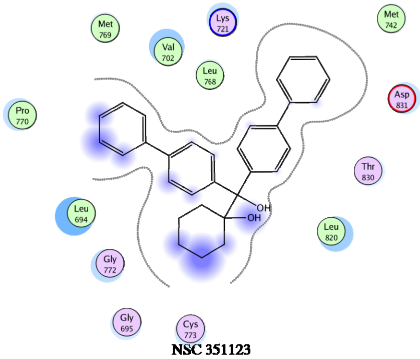

NSC 351123
Compound (L)

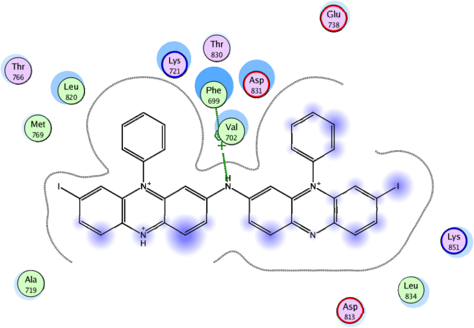

NSC 402959
Compound (M)

Table S1. The binding free energy and the score fitness value of the 3 known inhibitors from GOLD docking and scoring calculations.

Compounds	NSC no.	RMSD (Å)	RMSD (Å)	KCS score fitness value	GOLD Chemscore			ΔG(kJ/mol)	Average GI_{50} † (μg/ml)
		All heavy atom	Core structure		Hbond§	Lipo‡	DE Clash*		
Gefitinib	715055	3.67	0.81	30.05	0.70	229.62	0.26	-31.00	3.24×10-6
Erlotinib	718781	1.74	0.34	28.65	0.61	237.36	1.49	-31.03	5.51×10-6
AEE788	N/A	1.37	0.68	32.55	1.65	220.57	0.80	-34.04	N/A

†The average GI_{50} overall cell line from NCI Cancer Screen Current Data, May 2009. The GI_{50} means drug concentration causing 50% cell-growth inhibition; § and ‡ refer to protein-ligand H-bond and lipophilic contribution to the chemscore values, respectively, and * refer to protein-ligand clash penalty to the chemscore value.

S3. The core structure of the known inhibitors that used for calculate the RMSD values, the aniline-quinazoline group from gefitinib and erlotinib and the phenethyl-amino-pyrrolopyrimidine group from AEE 788.

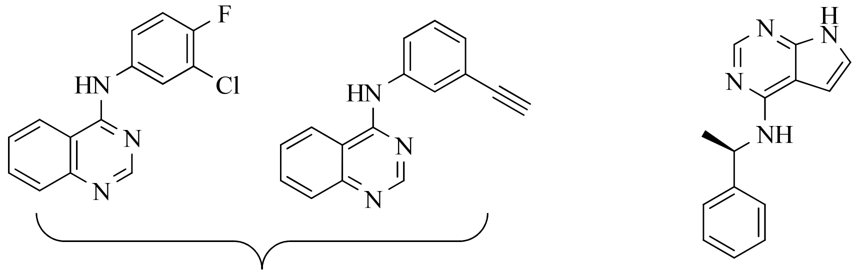

anilino-quinazoline group from gefitinib (left) and erlotinib (right)   core structure of AEE 788

© 2010 by the authors; licensee Molecular Diversity Preservation International, Basel, Switzerland. This article is an open-access article distributed under the terms and conditions of the Creative Commons Attribution license (http://creativecommons.org/licenses/by/3.0/).
